# International Journal of Implant Dentistry

**DOI:** 10.1186/s40729-014-0001-z

**Published:** 2015-02-12

**Authors:** Hendrik Terheyden, Yoshinobu Maeda

**Affiliations:** 1 Klinik für MKG-Chirurgie, Rotes Kreuz Krankenhaus Kassel gGmbH, Kassel, Germany; 2 Osaka University Graduate School of Dentistry, Osaka, Japan

Dear *International Journal of Implant Dentistry* reader,

Is there a place for a new electronic open access journal in implant dentistry?

We think there is. Here’s why:

- **Growing field:** The use of dental implants is increasing in most countries worldwide. Dental implants have become a safe and preferred therapeutic option in various indications. But more research still needs to be done.
- **Quick publishing:** Today the limitations of printed journals can be overcome by electronic publishing. With electronic journals there is no page limit and no need to wait for production deadlines. Articles can be published immediately after acceptance. The quality of the peer-reviewed articles becomes the most decisive factor for acceptance. Articles are published continuously over the year.
- **Rapid spread of information:** Readers can find articles in public search engines and download the full version of an article without charges. This ensures that information is spread quickly and may increase readership in a democratic way.
- **Creative Commons:** In many countries open access publications are mandatory for public research grants. Thus the information becomes freely available for the scientific community. In the open access structure of IJID, the article’s copyrights stay with the author and not with the publisher. The author can, for instance, reuse figures as long as the original source is cited correctly.
- **Publishing fees granted:** The German Association of Dental Implantology (DGI) and the Japanese Society of Oral Implantology (JSOI) pay the article publishing fees for the first four years. However, authors are kindly requested to include publishing fees in the grant applications for their future research projects.

The German Association of Dental Implantology (DGI) and the Japanese Society of Oral Implantology (JSOI) have decided to pool resources and work together synergistically. This journal initiative is an important part of this collaboration. Together both societies have more than 20,000 members and IJID readers.

On behalf of the entire editorial staff, we would like to thank you for your acceptance and utilization of this new publishing tool for your research and personal information.

